# Seizure network characterization by functional connectivity mapping and manipulation

**DOI:** 10.1117/1.NPh.12.S1.S14605

**Published:** 2025-01-16

**Authors:** James E. Niemeyer, Peijuan Luo, Carmen Pons, Shiqiang Wu, Hongtao Ma, Jyun-you Liou, Daniel Surinach, Suhasa B. Kodandaramaiah, Theodore H. Schwartz

**Affiliations:** a Weill Cornell Medicine, Department of Neurological Surgery, New York, United States; b The First Hospital of Jilin University, Department of Neurology, Changchun, China; c University of Chicago Medicine, Department of Neurosurgery, Chicago, Illinois, United States; d Tongji Hospital, Tongji Medical College, Hua Zhong University of Science and Technology, China; e Weill Cornell Medicine, Department of Anesthesiology, New York, United States; f University of Minnesota, Department of Mechanical Engineering, Minneapolis, Minnesota, United States; g University of Minnesota, Department of Biomedical Engineering, Minneapolis, Minnesota, United States; h University of Minnesota, Department of Neuroscience, Minneapolis, Minnesota, United States

**Keywords:** epilepsy network, seizure models, mesoscale imaging, extra-focal targeting, bilateral seizure propagation

## Abstract

**Significance:**

Despite the availability of various anti-seizure medications, nearly 1/3 of epilepsy patients experience drug-resistant seizures. These patients are left with invasive surgical options that do not guarantee seizure remission. The development of novel treatment options depends on elucidating the complex biology of seizures and brain networks.

**Aim:**

We aimed to develop an experimental paradigm that uses anatomical network information, functional connectivity, and *in vivo* seizure models to determine how brain networks, and their manipulation, affect seizure propagation.

**Approach:**

Guided by a known anatomical network, we applied widefield calcium imaging to determine how neural activity and seizures spread through the network regions, focusing on the primary somatosensory cortex and secondary motor cortex. We used *in vivo* microstimulation to induce suprathreshold excitatory activation and compared this reproducible stimulus with acute pharmacologically induced spontaneous seizure propagation. In a proof-of-concept experiment, we ablated a single node within this bilateral network and measured the effect on propagation and recruitment. Similar preliminary experiments were repeated in a chronic seizure model.

**Results:**

The microstimulation of the somatosensory cortex propagated in a distinct pattern throughout the bilateral network with sequential reproducible node recruitment. Seizures recapitulated this same pattern, indicating a hijacking of existing pathways. Ablation of a key node in the network in the secondary motor cortex changed contralateral spread. Early chronic cobalt seizure data are presented.

**Conclusion:**

Here, we demonstrate a paradigm for combining widefield calcium imaging with microstimulation, cortical ablation, and seizure mapping to determine how anatomical networks inform the propagation patterns of cortical seizures. These experiments can be extended to long-term tracking of epilepsy to study epileptogenesis in other cortical networks. Our proof-of-concept findings suggest that this paradigm may be useful in the development of novel therapies for drug-resistant epilepsy patients and can be extended to the study of other disorders involving brain networks.

## 1 Introduction

Epilepsy affects ∼1% of the world population at any time^1^ and shows a lifetime prevalence of 1 in 26 people.^2^ Despite the development and availability of various anti-seizure medications, more than 35% of epilepsy patients continue to experience seizures,^3^ leaving surgical options that typically target the seizure onset zone for disconnection, ablation, or neurostimulation. Yet, surgical options targeting this site do not guarantee seizure freedom,^4^^,^^5^ indicating that current concepts of seizure generation, which focus heavily on the seizure onset zone, are not fully adequate to explain its complex biology.^6^^,^^7^ Furthermore, in some circumstances, the seizure focus cannot be targeted because it overlaps with the functional cortex required for normal cognitive processing or behavior. These situations require alternative treatment strategies that do not ablate the seizure focus.

Epilepsy is increasingly considered a disorder of brain networks, where long- and short-distance axonal connections are believed to mediate the spread of seizures.^8^^–^^15^ Recent studies have highlighted how such connections are linked to seizure patterns in animals^16^^–^^19^ and humans,^11^^,^^20^^–^^23^ with researchers also demonstrating that abnormal white matter connections and brain network features are associated with surgical outcomes in epilepsy patients,^24^^–^^26^ indicating that regions outside of seizure onset zones may play critical roles in the development of seizures. Such widespread networks have even been shown to play a role in the suppression of focal interictal activity.^9^ Meanwhile, our lab recently reported that epileptiform activity propagation heavily depends on excitation/inhibition balance at distant brain sites.^27^^–^^29^ These findings all suggest a major role for widespread brain structure and local function in governing seizure initiation and propagation.

The overall aim of our research is to determine how networks inform seizure spread, with the goal of using this understanding to test novel treatment options and improve patient outcomes. Toward this aim, we have recently begun using mesoscale neural activity mapping to evaluate and examine brain networks in the context of seizure activity. With this work, we expect to determine the characteristics of brain networks and the relationship between nodes that underlie the spread of seizure activity. This is particularly important for understanding the nature of focal to bilateral tonic-clonic seizures, which cause loss of consciousness^30^ and carry an increased risk of death in human epilepsy patients.^31^

**Fig. 1:**
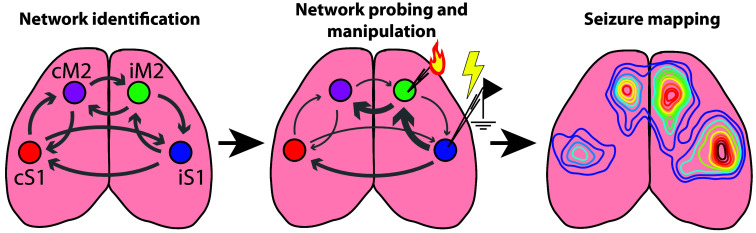
Executive summary of the paradigm. We first identified a neocortical network based on the Allen Institute mouse brain atlas. The chosen model network in this study was one involving primary somatosensory cortex (S1) and secondary motor cortex (M2). Next, we characterize how activity propagates through the sites of this model network following manipulations such as microstimulation and targeted cortical ablation. Finally, we examine how seizures, characterized by pathological hi-jacking of neuronal connections, can spread through this network before and after node ablation. All data presented in subsequent figures are preliminary but demonstrate the feasibility of this paradigm to study how neocortical nodes interact during pathological events and disease.

Our paradigm presented here includes several steps that, altogether, can provide an understanding of how the structural and functional connectivity of a brain network is related to the initiation and spread of seizures. A summary of our method is shown in Fig. 1; we used available Allen Institute virus tracing data to identify a neocortical network in the dorsal cortex of the mouse. This chosen network has previously been described by other groups performing widefield calcium imaging.^32^^,^^33^ We next applied cortical microstimulation during widefield calcium imaging to characterize how neural activity spreads through this model network, followed by cortical ablation of a cortical node to provide a proof-of-concept test of the importance of individual nodes in propagating bilateral brain activity. Finally, in preliminary data, we used an acute chemoconvulsant seizure model (4-Aminopyridine) to characterize how a focal seizure spreads within this network. The findings from this paradigm can provide an understanding of seizure networks and how they may respond to therapies that target network nodes outside the seizure onset zone.

## 2 Methods

Experimental procedures were approved by the Weill Cornell Medicine Institutional Animal Care and Use Committee following NIH guidelines.

### 2.1 Network Identification

**Fig. 2:**
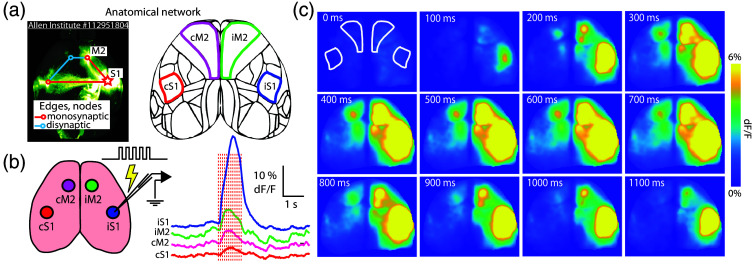
Bilateral S1-M2 network and functional mapping method. (a) Anatomical map of S1-M2 connectivity, provided by Allen Institute (experiment #112951804), highlighting bilateral connectivity of these neocortical network nodes. The schematic brain highlights the S1 and M2 regions. (b) The cartoon shows our stimulation paradigm used to measure functional connectivity during widefield calcium imaging. The right panel shows dF/F traces from bilateral S1-M2 nodes during stimulation of right S1. (c) Widefield imaging frames corresponding to vertical lines overlaid on traces in panel (b). Note the significant iM2 recruitment and clear cM2 recruitment, with a weaker signal appearing in cS1. White outlines on the first frame refer to the S1 and M2 nodes from panel (a).

We screened experiments presented by the Allen Institute mouse brain atlas and identified a neocortical network [Allen experiment 112951804, Fig. 2(a)] involving primary somatosensory cortex (barrel field, SSp-bfd) and secondary motor cortex (MOs). For brevity, we refer to these regions as S1 and M2. This network displays robust connectivity from S1 to ipsilateral M2 (iM2) as well as from S1 to contralateral S1 (cS1). Because of this bilateral connectivity, we also expected the involvement of contralateral M2 (cM2) in network activity. S1 coordinates used were (−1.7, +3.5) (AP, ML) relative to bregma; M2 coordinates (+1, +.75) (AP, ML). Dorsoventral values used in our experiments were always 300  μm below the cortical surface. These four nodes, iS1, iM2, cM2, and cS1, serve as the network components throughout our preliminary data shown in the analyses presented here. We note that, although we began our functional studies of this network based on the atlas anatomy, other previous reports have shown robust correlated activity in these regions.^32^^,^^33^

### 2.2 Animal and Cranial Window Preparation

All data were collected in C57-background mice expressing GCaMP in Thy1+ neurons (C57BL/6J-Tg(Thy1-GCaMP6f)GP5.5Dkim/J, Jackson lab #024276), referred to hereafter as Thy1GCaMP mice (across experiments, six mice were used, four male, two female, aged 8 to 10 weeks, with weights ranging 25 to 30 g). For cranial window implants, mice were first anesthetized with 3.5% isoflurane mixed with pumped ambient air. Body temperature was maintained at 37°C with an electric heating pad (Harvard Apparatus, Holliston, Massachusetts, United States). Once anesthetized, the mouse was head-fixed in a stereotactic frame (Kopf, Tujunga, CA, USA) with a nose cone delivering isoflurane at 1% to 1.5% throughout the surgery. Next, hair was removed with scissors, and a ∼8×9  mm opening was made in the skin over the skull. A craniectomy was performed over this region with a dental drill and size 005 bur. Next, an ∼8-mm circular window of PDMS material was placed over the exposed cortex and attached at the edges with Vetbond (3M, Hutchinson, Minnesota, United States). Then, a metal headbar (Neurotar, Helsinki, Finland) was implanted over this window and attached to the skull with C&B Metabond dental cement. Mice were given one injection of Meloxicam (NSAID) prior to surgery (0.5  mg/kg) and once per day for 3 days post-surgery.

### 2.3 Imaging

Mice were lightly anesthetized with 3% isoflurane for 30 to 60 s and then head-fixed by the Neurotar system (Neurotar, Helsinki, Finland). Widefield imaging was performed in the awake animal with a camera running at 120 Hz and frames being captured following the presentation of excitation light (470 nm) to induce GCaMP fluorescence. A 530-nm light was used to remove hemodynamic artifacts from the calcium imaging data elicited by 470-nm light. Both light wavelengths were presented by light-emitting diodes (LEDs) and optic fibers (Thorlabs, Newton, New Jersey, United States). The LEDs were time-locked to camera frame times. Further details can be found in Yang et al.^34^

### 2.4 Microstimulation

A glass micropipette with an ∼50-μm tip opening was backfilled with saline and placed over a stainless steel wire. This saline-filled electrode was attached to an amplifier (1000×) and then bandpass filtered (1 to 500 Hz) before signals were sent to a Cambridge Electronic Design (CED, Milton, Cambridge, United Kingdom) Power 1401 digitizer. This digitizer was attached to a PC running Spike2 (CED). The reference electrode was placed in the skin. Prior to microstimulation experiments, we first recorded brain signals (at 1 kHz) to verify that the electrode tip was fully inserted into the cortex. Next, we attached the electrode to the microstimulator system. This system is comprised of an ISO-Flex stimulus isolator (A.M.P.I, Jerusalem, Israel), which controls the current and polarity of stimulation and was set at 0.5 mA. This device itself was attached to a Master-8 controller system, which we programmed to elicit either 100-ms or 1-s stimulation trains of 1-ms pulses followed by 19 ms of rest, creating a 50-Hz stimulation. The stimulation was applied to the neocortex at right S1 and right M2 with 10 to 20 s between stimulation trials (100-ms S1 stim 31 trials, 1-s stim 51 trials; 100-ms M2 stim 22 trials, 1-s stim 60 trials). The stimulus train was sent to the CED system for alignment with simultaneously recorded imaging frames.

### 2.5 4-Aminopyridine Seizure Induction

An electrode was prepared as described above, except that instead of saline, we backfilled the pipette with 1-mM 4-aminopyridine (4-AP) in sterile saline.^34^ The electrode tip was then inserted through the PDMS into right S1 at a depth of 300  μm. 4-AP was then injected in 10-nL increments until focal seizures were induced, which typically occurred ∼100  nL. We note that, although seizures have been shown to propagate differently across cortical layers,^35^ our widefield imaging techniques do not permit such granular examination (although one would expect that layer 5 and L2/3 corticocortical projection neurons mediate the majority of the activity, we observe spreading across the S1-M2 corticocortical network). Seizures were monitored in Spike2 while performing widefield imaging. If seizures lasted longer than 10 min, then the experiment was stopped, and the animal was placed in the isoflurane induction chamber and anesthetized with isoflurane 3.5% for 10 to 20 min with a heating pad.

### 2.6 Thermal Ablation

We created a thermal electrolytic lesion to ablate a small region of the cortex following parameters described by previous ablation studies in rodents.^36^^,^^37^ A metal electrode (no pipette) was inserted into the right M2 in an anesthetized mouse following cranial window surgery. The electrode was coated with epoxy, and only the tip (0.5 mm) was exposed to the cortex. Using the ISO-Flex and Master-8 system, we delivered 1 mA of positive current for 10 s to the cortex. This amount and duration of current induced an immediate discoloration of the cortex around the electrode tip. The amplitude of stimulation was varied to create lesions of larger or smaller dimensions (see Sec. 3). Animals were provided meloxicam (0.5  mg/kg) after this procedure and recovered in a cage on a heating pad. Despite the visible cortical damage, no obvious symptoms were noted during the recovery period, and no adverse health issues (e.g., weight change) were noted. We expect that the lack of obvious symptoms was due to the damage in the association region of the brain; a similar lesion in the primary motor cortex, instead of M2, would likely produce changes in gait.

### 2.7 Mini-mScope Imaging of Spontaneous Cobalt Seizures

Cranial window surgery was performed as described above except that, following the craniectomy, a 0.5-mm length of cobalt wire (0.25-mm diameter) (Goodfellow, Pittsburgh, Pennsylvania, United States) was inserted into right M2. We also use a PET window mounted on a 3D printed frame, rather than the PDMS film window,^38^^,^^39^ to connect with a widefield microscope. This harder PET material does not permit injections through the window, which are not needed for the cobalt wire model, and we find that it reduces reflections detected by the CMOS mesoscope camera. A thorough description of the mini-mScope system can be found in Rynes et al.^40^ Briefly, this head-mounted camera uses the CMOS sensor in the miniscope platform^41^^–^^43^ and is capable of performing widefield calcium imaging across the whole dorsal cortex.^40^^,^^44^^,^^45^ The camera and LED housing are magnetically attached to the implanted head bar on the mouse. This lightweight device (3.8 g) is tethered to a small commutator to permit a 360-deg rotation during animal movement (also see Ref. 44). Two LEDs deliver 470- and 530-nm wavelengths for GCaMP excitation and hemodynamic correction, respectively. The 470-nm light, in our system, is presented for 20 ms followed by 530-nm light for 4 ms, and frames are captured at 30 Hz. Data are saved to disk in.avi format and subsequently analyzed in MATLAB using the same methods as our head-fixed widefield imaging system. The commutator is attached to the removable roof of a clear plexiglass box (12×12×12 inches), which contains bedding material, water, and food. After attaching the camera to the awake mouse, we can record continuously for up to 6 h. Seizures develop in most mice within 1 day after cobalt insertion but greatly decrease by day 4,^46^^–^^49^ so imaging is performed on post-implant days 1 to 3. Seizure data are then analyzed offline.

### 2.8 Imaging Data: Processing and Analysis

All data analysis was performed in MATLAB using custom-written scripts. Calcium imaging data were purified by separation of slow hemodynamic artifacts^34^ by the equation:

$$\frac{F\text{true}(t)}{F\text{true}(tb)}=(\frac{F(t)}{F(tb)})/(\frac{I(t)}{I(tb)}),$$

where Ftrue(t) is the purified GCaMP signal after removing the hemodynamic bias at frame or timepoint (t). F is the fluorescence detected, tb is the baseline time, and I is the intrinsic optical signal observed at 530 nm following 2-Hz lowpass filtering. Change in fluorescence, dF/F, was calculated by taking a baseline time immediately (1 s) preceding microstimulation trials or seizures

$$\frac{dF}{F}=\frac{Ft-Fb}{Fb},$$

where Ft is the fluorescence at frame t (after hemodynamic correction), and Fb is the baseline fluorescence. Temporal smoothing was then applied by using a three-frame (50 ms) moving average. Correlation maps following microstimulation were created by first taking the dF/F at the stimulation site and then calculating the Pearson’s correlation coefficient (r) between that dF/F trace and the dF/F trace from all other pixels in the field of view in a time window of 2 s after the stimulation. We calculated the area under curve (AUC) of the dF/F response at individual nodes by taking the average activity of pixels in a 3×3 region centered on the anatomical node site, also in a 2-s window after the stimulation, using the trapz() function in MATLAB to calculate the AUC.^50^ Maximum dF/F activity was also taken from this period. Time to peak response was calculated by finding the onset, defined as the peak of the second derivative, and then measuring the number of frames until reaching the peak response. Decay responses were calculated by finding the peak and then calculating the AUC until the end of the 2-s window, to calculate a value of “lastingness” of the response after the stimulation.^51^ To account for small variations in calcium responses between stimulations and animals, normalization was performed by dividing the AUC or maximum activity at the stimulation site. Thus, the microstimulation population data show responses relative to the stimulation site on a within-trial basis. Two-dimensional data that are shown in figures and videos were spatially smoothed frame-by-frame with a two-sigma Gaussian kernel (imgaussfilt in MATLAB) to reduce artifacts from blood vessels. Node regions were identified by stereotactic marking of bregma made during the cranial window implant surgery. Mini-mScope data were analyzed in the same manner as the head-fixed imaging data, albeit at a 30-Hz overall frame rate rather than 120 Hz. Microstimulation videos were created by averaging trials within one mouse: this was achievable because stimulation patterns were identical (1 s of 50 Hz) across trials.

### 2.9 Seizure Propagation Analysis

To map the direction of seizure propagation, we adapted the method of line length, commonly used in EEG seizure analysis and detection.^13^^,^^52^^–^^54^ We treat each pixel in the field of view as a signal source akin to an electrophysiology channel and use a moving 5-s window to calculate the cumulative amount of change in dF/F following high-pass filtering (f>1  Hz). Using a baseline from a period of 5 s before seizure onset, we then determine when each pixel crosses a threshold of 2× baseline line length. This is repeated for each pixel in the field of view to determine the time at which the pixel is recruited to the seizure. The onset times for each pixel are then aggregated together in a heatmap to produce a spatiotemporal description of seizure spread. For clarity, two-dimensional Gaussian filtering (kernel sigma = 2 pixels) is applied to the resulting map data.

## 3 Results

After reviewing the Allen Institute atlas of synaptic connections, which is based on viral tracing in the mouse brain (mouse connectivity atlas experiment #112951804^55^), we chose the S1 and M2 regions as a model bilateral network to study, treating S1 as a focus in an epilepsy network. S1 is connected monosynaptically with M2. Each of these sites is also connected monosynaptically with their contralateral counterparts (cS1 and cM2). In a recent study, we examined how bicuculine-induced interictal activity propagates through this network,^27^ observing clear propagation of activity through each of the four bilateral nodes that have been previously shown to display correlated network-like activity.^32^^,^^33^

The second step of our paradigm is to characterize the functional connectivity of this anatomical network. To achieve this, we applied microstimulation to the primary somatosensory cortex during widefield calcium imaging of Thy1GCaMP mice. Following microstimulation of S1, network node activation is observed (Fig. 2). Outside of the stimulation site, the strongest response occurs in ipsilateral M2 (iM2), followed by contralateral M2 (cM2), and then contralateral S1 (cS1), with some recruitment of medial brain regions (e.g., retrosplenial cortex). Figure 2 shows the activity profile following 1-s stimulation (50 Hz, 0.5 mA) of right S1.

**Fig. 3:**
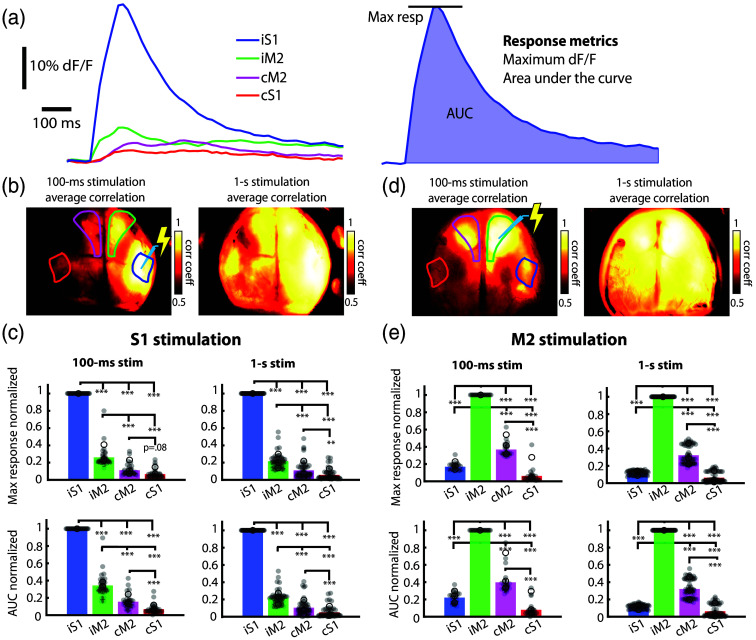
Microstimulation highlights the strength of M2 bilateral connectivity. (a) Response traces from one trial of 100-ms stimulation of S1. The image at right shows the two measures of responsiveness: maximum response (normalized to the peak response at the stimulation site) and the AUC of the response (normalized to the AUC at the stimulation site). (b) Example correlation maps from one mouse, calculated for 100-ms and 1-s stimulations. S1 and M2 regions are shown in colors that are associated with bar colors below. (c) Maximum and AUC responses to 100-ms and 1-s stimulations of S1. (d) Example correlation maps from one mouse, calculated for 100-ms and 1-s stimulations. (e) Maximum and AUC responses to 100-ms and 1-s stimulations of M2. In all plots, gray circles represent individual trials and open circles show averages for the N=3 mice. Error bars represent +/− SEM.

To understand how activity propagates through the S1-M2 network, we next applied microstimulation, in separate experiments, to both S1 and M2 during widefield imaging and quantified the propagation of activity through this network (Fig. 3). Figure 3(a) shows activity traces in response to stimulation and a diagram of the response measurements taken. Correlation maps are also shown (seeded at the stimulation site) for both S1 and M2 stimulations [Figs. 3(b) and 3(d)]. Figures 3(c) and 3(e) show signal quantifications for two of these stimulus durations using two metrics: maximum response and the AUC, both normalized within the trial to the response at the stimulation site. Following stimulation, each region of the bilateral S1-M2 network shows a significantly different response from every other node (N=3 mice, one-way ANOVA with Tukey’s honestly significant difference (HSD) test, ***p<0.001, **p<0.01, *p<0.05). The strongest activity was always observed at the stimulation site, but outside of the stimulation site, the next strongest activity appeared at ipsilateral M2. Interestingly, despite direct connectivity between iS1 and cS1, there was a consistently significantly stronger response at cM2 compared with cS1.

This finding suggests that cross-callosal excitatory connections may be more robust between iM2 and cM2 compared with iS1 and cS1. To rule out the possibility that the activation by the stimulating electrode, compared with naturally propagated activity, was suppressing the contralateral homotopic node, we next applied microstimulation at right M2. Correlation maps [Fig. 3(d)] show that stimulation of M2 strongly recruits contralateral M2 with high fidelity. When examining the AUC and maximal responses, we observed that the activity in cM2 was about twice as strong as the responses at iS1 and cS1 [Fig. 3(e)]. As with S1 stimulation, all nodes showed significantly different responses from each other (N=3 mice, one-way ANOVA with Tukey’s HSD test, ***p<0.001, **p<0.01, *p<0.05).

Our stimulation data showed a strong functional connection between iS1 and iM2 and an even stronger functional connection between iM2 and cM2. Meanwhile, cS1, although clearly responsive to stimulation and appearing as a node in the network, was always the weakest responder. These preliminary findings suggest a biased pathway for excitatory neural activity: the strongest being a cross-callosal connection between iM2 and cM2, followed by the within-hemisphere connection from iS1 to iM2 and then the weakest connection being the cross-callosal connection between iS1 and icS1. These findings strongly suggest that there is marked anatomic variability in the strength of excitatory cross-callosal connections with motor-frontal connections being robustly excitatory and sensory connections only weakly so. Future experiments that employ this network stimulation paradigm, using more animals, will provide a more comprehensive understanding of this connectivity.

**Fig. 4:**
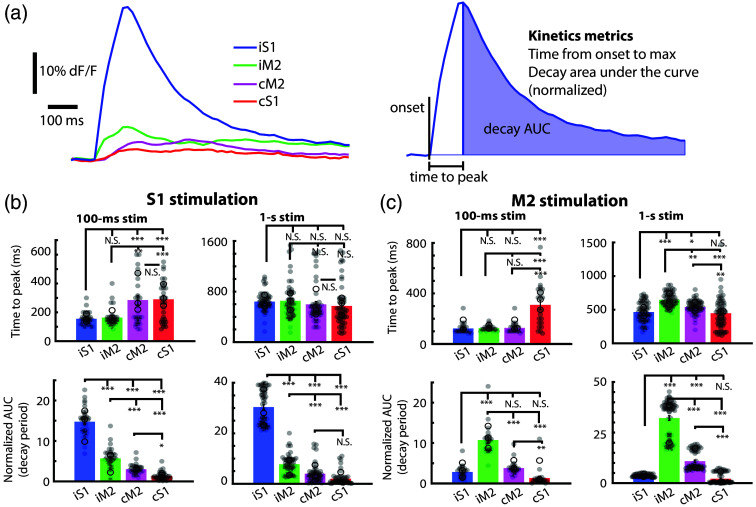
Kinetics of response across cortical nodes following stimulation. (a) Response to 100-ms stimulation and pictorial description of the two response kinetics measurements: Time to peak, and the AUC during response decay. (b) Time to peak and AUC during response decay for 100-ms and 1-s stimulations of iS1. (c) Time to peak and AUC during response decay for 100-ms and 1-s stimulations of iM2. In all plots, gray circles indicate individual trial data, open circles represent within-animal averages, and error bars denote +/− SEM.

We next examined the kinetics of network responses to microstimulation at S1 and M2 nodes (Fig. 4). We calculated the time to peak response at each node as well as the AUC during the decay period, a measure of the “lastingness” of the signal.^51^ Overall, we observed a wide variation in response kinetics but found that the S1 stimulation site and ipsilateral nodes both showed significantly different onsets (100-ms stim, iS1 versus cM2, cS1 p<0.001, iM2 versus cM2 p<0.01, iM2 versus cS1 p<0.001, one-way ANOVA with Tukey’s HSD, N=3 mice), although not for 1-s stimulations. Interestingly, the 1-s stimulation showed notable variability in the time to peak, suggesting that a shorter 100-ms stimulation may be more effective at mapping network connections. The AUC during decay meanwhile showed a robust difference between all nodes in a manner very similar to the initial response measurements [Figs. 3(c) and 3(e)], such that the AUC during decay at each node was significantly stronger than the activity at each downstream node [Fig. 4(b) bottom, one-way ANOVA with Tukey’s HSD, N=3 mice]. Stimulation of M2 resulted in similar effects in onset kinetics: 100-ms stimulation showed a significant difference between iM2 and cS1 (p<0.001) but not other nodes. Stimulations of 1 s were significant across all node comparisons of onsets [Fig. 4(c), right top] excepting iS1 versus cS1, though the variability in this 1-s stimulation condition is notable. The AUC during response decay following iM2 stimulation was significantly weaker at all other node sites, following the trend of the S1 stimulation such that more distant nodes showed lower decays. This effect was present following both 100-ms and 1-s stimulations [Fig. 4(c)].

**Fig. 5:**
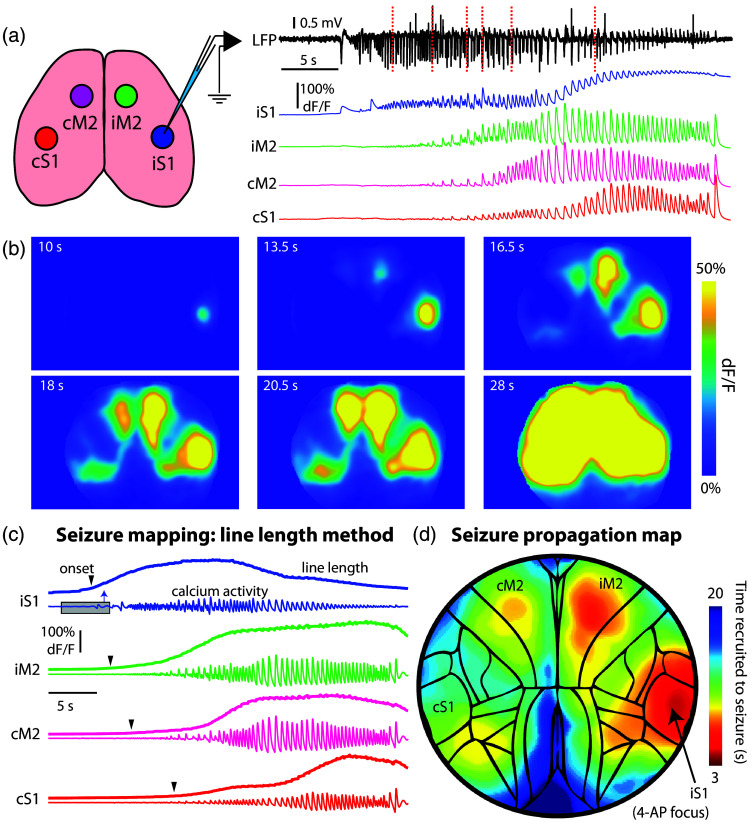
Method for mapping seizures in a neocortical network. (a) 4-AP is injected into right S1, which induces robust focal seizures that propagate across the cortex. Traces show electrophysiology from the ictal focus (black) and calcium imaging data (dF/F) from each of the bilateral S1-M2 network nodes. (b) Calcium imaging frames are shown from the times corresponding to red dotted lines in panel (a). Note that not all S1-M2 network nodes are recruited simultaneously but are invaded successively over many seconds. (c) We determine seizure spread by using an adapted line length method, calculated in a moving 5-s window of calcium activity following low-frequency removal to determine, for each pixel in the field of view, when seizure recruitment occurs (arrows). (d) The result of our line length method is a heatmap showing the time of seizure onset for each pixel, creating a single map of the propagating seizure in this bilateral network.

A major component of our paradigm is to examine how seizure propagation correlates with the functional connectivity observed in the above experiments. Following this mapping, we next examined how pharmacologically induced seizures propagate through the bilateral S1-M2 network. After injecting 4-AP at the S1 node, we tracked seizure propagation with GCaMP imaging in one mouse. Individual imaging frames, and simultaneously measured electrophysiology, show the ictal development over time (Fig. 5). To visualize this event as a single map, we applied an adapted line length method, calculated in 5-s windows, to the calcium data to determine when the seizure invaded each pixel in the field of view. Although line length has become a standard metric in EEG seizure detection,^13^^,^^52^^–^^54^ its application in calcium imaging is new and arguably a significant improvement over standard metrics that depend solely on dF/F increases. Unlike thresholded dF/F values, the line length provides a quantification of the change in signal over time, which we calculate in a moving time window. Thus, this method adds to other recently developed methods for seizure detection using calcium signals.^29^^,^^56^^–^^58^ As seen in Fig. 5, this method provides a clear description, and single heatmap, of where the seizure begins and when it recruits other neocortical regions. In agreement with our microstimulation experiments, the seizure propagates through the bilateral S1-M2 nodes. Similar to microstimulation experiments, the recruitment of cM2 appears more robust than the recruitment of cS1, despite a strong monosynaptic connection between iS1 and cS1. This finding implies that seizures may preferentially spread across specific callosal pathways and heterogeneously across the callosum as a whole.

Based on our findings that anatomical networks, determined from axonal connections observed by viral tracing experiments, can inform neural activity (stimulation) and seizure propagation, we next sought a method to test how the manipulation of network nodes could affect the spread of this activity. Although most surgical interventions in human epilepsy will target the seizure onset zone, various studies have also suggested that manipulation of network components outside of the onset zone may also serve as treatment targets.^6^^,^^12^^,^^13^^,^^59^^–^^61^ Given the robust role of iM2 in the S1-M2 network, in both microstimulation and seizure experiments, we chose to ablate this extrafocal region. Under anesthesia, we inserted a microelectrode through the PDMS film window and brought the tip into the secondary motor cortex, choosing coordinates based on microstimulation. With the electrode tip in M2, we then passed 1.0 mA of current through the electrode for 10 s; parameters that we have found will robustly destroy neural tissue within 0.5 mm of the tip as visualized from the surface of the brain (experiments have not yet been performed to determine the 3D extent of tissue damage following ablation, but this finding is in agreement with others using similar parameters in rodents^36^^,^^37^).

**Fig. 6:**
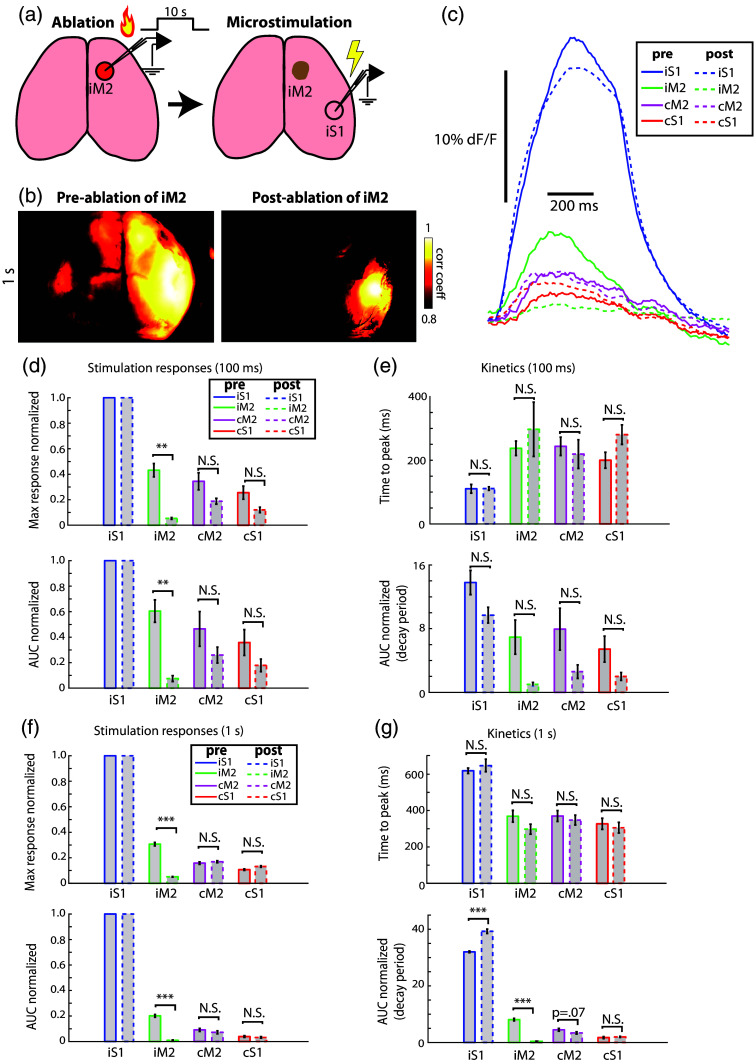
Proof of concept: node ablation alters activity propagation through the bilateral S1-M2 network. (a) Thermal ablation was applied to right M2, followed by microstimulation in iS1 in one mouse. (b) Panels show example correlation maps, seeded at the S1 stimulation site for 1 s stimulation before and after ablation (pre-ablation, Video 1; post-ablation, Video 2). (c) Traces of node activity are shown for pre- and post-ablation (same animal) during 1-s microstimulation. (d) Stimulation responses, maximum and AUC, are shown for pre- and post-ablation in one test animal. (e) Kinetics of responses, time to peak, and AUC during the decay period, are shown for pre- and post-ablation in one test animal. (f), (g) same as (d) and (e) but for 1-s stimulation periods. In all plots, error bars represent +/− SEM (Video 1, Mp4, 560 KB [URL: https://doi.org/10.1117/1.NPh.12.S1.S14605.s1]; Video 2, Mp4, 566 KB [URL: https://doi.org/10.1117/1.NPh.12.S1.S14605.s2]).

Three days after ablation, we then performed a microstimulation experiment again to examine how neural activity spreads following this network disruption. Figure 6 shows how microstimulation activity at iS1 propagates before versus after ablation of iM2 [Figs. 6(a)–6(c)]; Videos 1 and 2). As expected, the activity from iS1 did not spread clearly to the centroid of iM2 but rather spread only to the margins of this region. Meanwhile, the correlation of cM2 and iS1 was reduced. We also measured response characteristics pre- and post-ablation [Figs. 6(d)–6(g)]. Robust differences were seen in both metrics at iM2 following ablation, as expected (Wilcoxon rank sum test, ***p<0.001, **p<0.01, *p<0.05), except for kinetics measurements where only the 1-s stimulation showed a significant effect in this mouse. This is presumably a result of using a normalized kinetics measurement on data from an ablated brain region. Interestingly, following the ablation of iM2, we also observed a trend of reduced response differences in cM2, but the general trend across comparisons was only a small (non-significant) change in the activity at cM2. Subsequent experiments with more animals will focus more closely on the strength and timing of activity following node ablation.

**Fig. 7:**
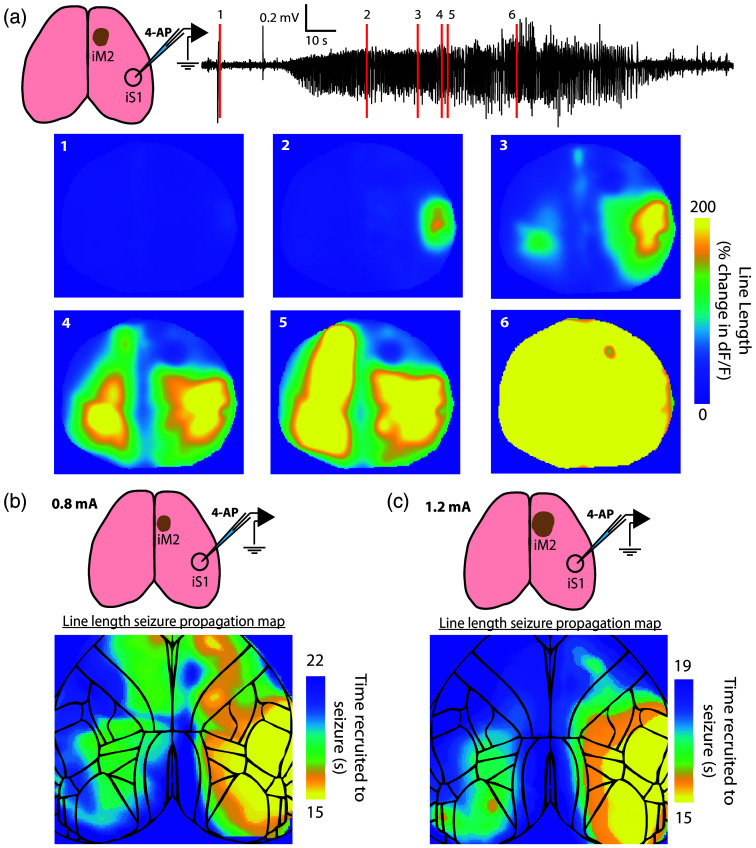
Preliminary test of ablation on seizure patterns. (a) In one mouse, a 1-mA current injection for 10 s was used to ablate the right M2. A seizure, including its associated line length, is shown, highlighting how M2 is no longer recruited to the seizure (though, by late stages, fluorescence activity encroaches considerably from the edges of the lesion site). (b) Following a 0.8-mA lesion of iM2, a seizure was initiated and mapped in one animal. Note that seizure propagation still occurs across the corpus callosum at anterior M2 sites. (c). Following 1.2-mA lesioning of iM2, a seizure was initiated and mapped in the same animal. Note that the seizure is delayed in crossing the anterior corpus callosum and crosses into the rostral cortex (visual and visual association cortices) before reaching cM2.

We next examined how seizure propagation can be altered by a similar M2 node ablation in preliminary data. Figure 7(a) shows a 4-AP seizure experiment performed following M2 ablation in one mouse. The black trace shows the LFP recorded at the 4-AP seizure focus, and the red lines indicate the times of imaging frames below. Frames show the line length measurement of seizure spread and demonstrate that this seizure did not spread to iM2 until all other dorsal cortical regions had been recruited to the seizure. Furthermore, cM2 was recruited following cS1, unlike the standard pattern of seizure spread in this network (cf. Fig. 5). We also tested the importance of electric current level in node ablation. Figure 7(b) shows seizure propagation maps following 0.8-mA current ablation at iM2 (left panels) in one animal. Note that, like the microstimulation data, activity still propagated to the edges of iM2, but due to ablation, the seizure did not spread directly to iM2. This finding indicates that, despite thermal ablation at the node center, some neuronal activity may remain functional at the perimeter of the ablated territory. We thus reasoned that a larger ablation would more significantly alter the seizure propagation and therefore performed a second ablation with 1.2 mA of electric current [Fig. 7(c)]. This 50% increase in electric current resulted in a larger region of ablated tissue as seen from the cortical surface. When seizures were later initiated by 4-AP at iS1, we observed a change in the propagation patterns [see seizure maps, Figs. 7(b)–7(c)]. Although some seizure activity does still invade the edges of iM2, the seizure invades cM2 only after spreading contralaterally at a site somewhat medial and posterior to cS1. In other words, when sufficiently ablated, iM2 no longer serves as a propagation node to cM2. Thus, we have successfully changed the seizure propagation by manipulating a node within the seizure network without altering the seizure focus itself.

**Fig. 8:**
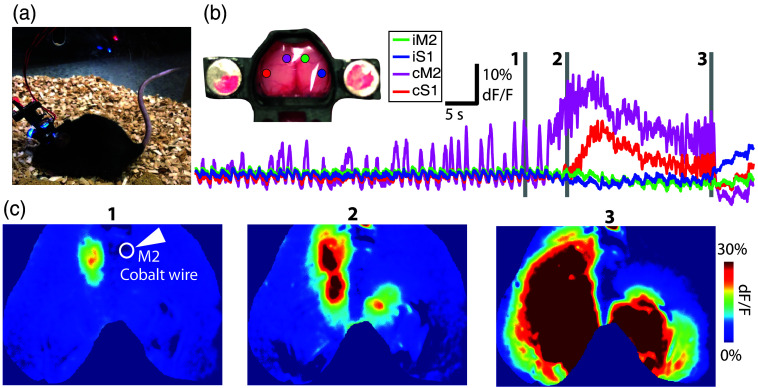
Methods for capturing spontaneous seizures. (a) Mini-mScope camera attached to an awake behaving mouse implanted with cobalt wire in right M2. (b) Mini-mScope implanted headmount showing brain with S1-M2 network nodes, and associated imaging data from these nodes during a spontaneous seizure. Note that the data do not show initiation at the cobalt insertion site but rather at cM2. (c) Mini-mScope imaging frames, times denoted by gray lines in panel (b), showing the contralateral development of the seizure. Note that there is no time during the seizure when iM2, the cobalt insertion site, shows changes in calcium activity reflecting the seizure.

To extend our paradigm and findings from an acute pharmacological model to a chronic model of spontaneous recurring seizures, more akin to human epilepsy, we have begun optically probing seizures in the cobalt wire model of epilepsy.^62^^–^^65^ Due to the low frequency of seizures in this model, we have turned from head-fixed imaging to the novel mini-mScope, a head-mounted widefield imaging system that permits the free behavior of a mouse (see Sec. 2.7, Methods) and imaging for several hours at a time.^40^^,^^44^^,^^45^ Figure 8 shows preliminary data collected from a spontaneously occurring seizure 1 day after cobalt insertion into the right M2 of a Thy1GCaMP6f mouse. M2 was chosen for initial tests because of its known ability to produce seizures in chronic epilepsy models.^62^^,^^66^^,^^67^ Note that the seizure does not appear in the calcium data at the insertion site. Although electrographic studies have shown that cobalt induces seizures at the insertion site,^46^^,^^48^^,^^49^ our seizure presented here suggests that damage to that site may prohibit the ability to capture calcium changes (see Sec. 4, Discussion). We are still in the early stages of investigating this phenomenon and characterizing the cobalt seizure network, which will vary based on the anatomic site of seizure initiation. However, we expect that this new free-behaving system will provide a next-step addition to the paradigm presented in this study.

## 4 Discussion

The neocortex is comprised of various interconnected networks, characterized by large groups of neurons and their axonal connections across large regions of the brain. Although these networks are now generally accepted as mediators of seizures and interictal activity,^12^^,^^13^^,^^27^^,^^68^^,^^69^ the basic mechanisms underlying the initiation and spread of this activity through different brain sites are not understood.

Here, we presented a paradigm composed of sequential methods for studying neocortical networks underlying seizures in rodent models of epilepsy that may be useful for examining these mechanisms. We are particularly interested in determining how secondary generalization of focal neocortical seizures is affected by nodes of the brain that are physically distant from the seizure onset zone. By choosing a network based on known anatomical connections, such as between S1 and M2, we can examine the propagation patterns of neural activity and seizures. In our preliminary experiments with this paradigm, we showed that activity propagates robustly from iS1 to iM2, with significantly more strength than to cS1. This finding was unexpected because cS1 and iM2 are both known to share monosynaptic connections with iS1. Furthermore, we find that neural activity is most likely to cross to the contralateral cortex at the anterior M2 site. We evaluated this with microstimulation, but the effect also appears in 4-AP-induced chemoconvulsant seizures, suggesting that the anterior M2 corpus callosum projections may permit significantly more activity transfer than the S1-cS1 callosal connections. In agreement with this, when we applied microstimulation to right M2, we observed a robust recruitment of contralateral M2. These preliminary findings suggest a major role for anterior M2 in the propagation of seizures and a marked heterogeneity in cross-callosal connections.

In recent years, laser interstitial thermal therapy (LITT) has become available in the clinic as a technique for the treatment of medically intractable epilepsy. Mostly employed to treat mesial temporal lobe epilepsy, hypothalamic hamartomas, and periventricular heterotopias, in principle, LITT could also be used to ablate critical seizure propagation nodes when the seizure focus resides in the functional cortex. We investigated the possibility of such a therapy in the S1-M2 network by targeting the M2, rather than the S1 node. In our proof-of-concept experiment, we found that ablation of iM2, a key node in the S1-M2 network, attenuated the propagation of suprathreshold electrical stimulation in S1. As expected, ablation of iM2 also delayed and attenuated seizure spread when the focus was in iS1. However, in our pilot experiments, it appeared that the seizure found an alternate pathway to recruit the contralateral hemisphere through a more posterior site (around the visual cortex) before spreading anteriorly to cM2. This preliminary finding does not, by itself, indicate that extrafocal network manipulations are incapable of inhibiting seizures. The ablation of M2 in our experiment only serves as a proof-of-concept that seizure spread can be changed by node knockdown, and further studies that use cell-type-specific manipulations or combined callosal and nodal ablations will provide a better understanding of how an extra-focal node manipulation may impact seizure spread. For example, using DREADDs,^70^^–^^72^ optogenetics,^73^^–^^76^ or magnetogenetics^77^ with cell-type specificity to increase inhibitory tone or decrease excitatory tone (or both) in iM2 may more effectively impede seizures.

We chose an anatomical network that permitted widefield calcium imaging and network manipulation. However, it is not clear which findings from our network are generalizable to other brain networks. For example, we observed that contralateral seizure propagation occurs at the anterior M2 site when the seizure originates in S1; however, a seizure originating in a different site may spread contralaterally across a different callosal connection. This may be particularly relevant when generalizing this neocortical network to hippocampal seizures, which may be more likely to spread across the hippocampal commissure and recruit different neocortical sites. Furthermore, our work here does not currently address the role of other subcortical network nodes. The ventral posteromedial nucleus of the thalamus (VPM), a subcortical component of this network,^32^^,^^33^ is robustly interconnected with rodent S1. In a recent study, we showed that the inhibition of the VPM by tetrodotoxin can reduce the dF/F observed in the ipsilateral cortex,^78^ but we do not yet know how this network node with its thalamocortical loop may affect the initiation and propagation of S1 seizures. Future experiments will test how ablation or inhibition of VPM can impact seizures.

Recent reports have suggested that seizure control may be achievable by targeting regions of the brain outside of the seizure focus for neurostimulation. For example, in assessments of seizure reduction in patients who received brain-responsive neurostimulation (RNS) for medial temporal lobe epilepsy, it was found that the stimulation need not be applied directly in the hippocampal focus to achieve a reduction in epileptiform activity.^79^^,^^80^ In further agreement with a role for extra-focal nodes in epilepsy, one recent study reported that human focal interictal activity is actively suppressed by other brain regions,^9^ which was proposed as the reason that interictal spikes do not always result in epileptic seizures. The development of extra-focal epilepsy therapies^12^^,^^13^^,^^60^^,^^81^ will be enhanced by laboratory studies using paradigms such as ours to understand how different regions interact during seizures.

Using the mini-mScope,^40^ our initial tests of the cobalt wire model of spontaneous recurring seizures highlight a possible difference in calcium imaging of chemoconvulsant-induced and spontaneous seizures. In imaging, our 4-AP seizures clearly develop at the site of injection before propagating. Meanwhile, the example seizure induced by cobalt wire appears to initiate in the contralateral cortex. Whether this difference is due to the seizure onset zone being S1 (4-AP) versus M2 (cobalt) is unclear. However, the cobalt seizure never appears in the calcium imaging at the site of cobalt insertion. The fact that the putative seizure onset zone, as determined in electrophysiology studies,^46^^,^^49^^,^^62^^,^^63^^,^^66^ never shows seizure activity suggests that the focal damage of the wire (and subsequent inflammatory response) may prohibit calcium imaging at this site. An inability to perform widefield calcium imaging of the seizure focus in a damaging model could be a problem for our mesoscale paradigm; however, we expect other epilepsy models, such as ones that rely on non-damaging genetic alterations,^82^ optogenetics,^83^^,^^84^ or DREADDs,^85^ to overcome this problem.

## 5 Conclusion

Brain networks undoubtedly serve a role in epileptic seizures, and mesoscale methods that employ widefield, bilateral, calcium imaging can uncover useful information about how seizures start and spread. Our paradigm and preliminary data presented here show a pathway that can be used to determine how activity spreads through interconnected brain regions in healthy conditions and disorders such as epilepsy. By probing how different network nodes interact during seizures, we expect to find new actionable targets, such as extra-focal brain sites and connections, that can be manipulated to prevent or inhibit seizures. Extending this paradigm into chronic models that better emulate human disease, along with free-behaving imaging technologies, will allow the study of longer-term disease development and network re-organization in addition to acute seizures.

## Supplementary Material





## Data Availability

Data are available from the authors upon request.
